# Boosting membrane carbon capture via multifaceted polyphenol-mediated soldering

**DOI:** 10.1038/s41467-023-37479-9

**Published:** 2023-03-27

**Authors:** Bin Zhu, Shanshan He, Yan Yang, Songwei Li, Cher Hon Lau, Shaomin Liu, Lu Shao

**Affiliations:** 1 State Key Laboratory of Urban Water Resource and Environment, School of Chemistry and Chemical Engineering, Harbin Institute of Technology, Harbin, China; 2 Key Laboratory of Materials Processing and Mold (Ministry of Education), National Engineering Research Center for Advanced Polymer Processing Technology, Zhengzhou University, Zhengzhou, China; 3 School of Engineering, The University of Edinburgh, Edinburgh, UK; 4 WA School of Mines: Minerals, Energy and Chemical Engineering, Curtin University, Perth, Australia

**Keywords:** Chemical engineering, Organic-inorganic nanostructures

## Abstract

Advances in membrane technologies are significant for mitigating global climate change because of their low cost and easy operation. Although mixed-matrix membranes (MMMs) obtained via the combination of metal-organic frameworks (MOFs) and a polymer matrix are promising for energy-efficient gas separation, the achievement of a desirable match between polymers and MOFs for the development of advanced MMMs is challenging, especially when emerging highly permeable materials such as polymers of intrinsic microporosity (PIMs) are deployed. Here, we report a molecular soldering strategy featuring multifunctional polyphenols in tailored polymer chains, well-designed hollow MOF structures, and defect-free interfaces. The exceptional adhesion nature of polyphenols results in dense packing and visible stiffness of PIM-1 chains with strengthened selectivity. The architecture of the hollow MOFs leads to free mass transfer and substantially improves permeability. These structural advantages act synergistically to break the permeability-selectivity trade-off limit in MMMs and surpass the conventional upper bound. This polyphenol molecular soldering method has been validated for various polymers, providing a universal pathway to prepare advanced MMMs with desirable performance for diverse applications beyond carbon capture.

## Abstract (web summary)

The development of mixed-matrix membranes is especially challenging when highly permeable materials are used. Here the authors present a molecular soldering strategy featuring multifunctional polyphenols in tailored polymer chains, well-designed hollow MOF structures, and defect-free interfaces to break the permeability-selectivity trade-off limit.

## Introduction

Persistently high level of atmospheric carbon dioxide (CO_2_) is a crucial cause of ruinous climate change. In addition, it hinders the Paris Agreement’s temperature targets for global CO_2_ net emission to be cut in half by 2030 and reach zero by 2050^1^. Carbon capture and sequestration (CCS) is a reliable mitigation approach for meeting these goals^2,3^. The membrane-based gas separation technique is an inherently energy-efficient solution for carbon capture, offering several prominent advantages, such as cost-effectiveness, small footprint, environmental friendliness, and easy operation^4–6^. The development of various membrane modules has attracted research attention, among which advanced polymeric materials remain predominant in the market owing to their favorable processability, mechanical robustness, and low cost^7–9^. However, the intrinsic trade-off between the gas permeability and selectivity of conventional polymer membranes severely limits their separation performance and hampers their broad applications^10,11^.

The emergence of the mixed-matrix concept has provided a potential platform to overcome the trade-off limit, wherein inorganic particles with superior separation capacity are incorporated into a processable polymer matrix^12,13^. However, interfacial matching during the rational design of mixed-matrix membranes (MMMs) remains challenging. The mismatch at the interface between the polymer matrix and fillers increases the number of nonselective voids and decreases selectivity^14–17^. The recognized solution to improve interfacial compatibility is to enhance the polymer–filler interaction through surface functionalization, morphology regulation, ligand exchange, and other approaches^18–21^. While a strong interaction in MMMs, especially in highly permeable ladder polymers such as polymers of intrinsic microporosity (PIMs)^22,23^, adversely affects the porosity and stiffness of the surrounding matrix. Although such rigid surfaces may help improve selectivity and impede physical aging of the PIM owing to the arrangement of polymer chains, they also result in a pronounced free volume loss and decreased permeability.

Metal-organic frameworks (MOFs), consisting of metal ions/clusters linked by organic ligands, are perceived as one of the most promising fillers in MMMs for sub-nanoscale gas separation owing to their unique topological structure^24–26^. Previous studies have focused on improving the gas separation performance by incorporating different MOFs into a polymer matrix. However, the poor compatibility between the two components induces an undesirable effect on membrane separation performance^27^. In addition, the ideal MOF structures for MMMs should possess not only proper molecular sieving ability or favorable gas adsorption capacity but also moderate mass transfer resistance to meet the expectations set by an endless array of high-performance polymer materials that have substantially pushed the CO_2_ permeability to the level of 10^3^ Barrer. Hollow MOFs offer unique structural benefits and provide a high fraction of free volume for gas transport. Therefore, it is expected that the enhanced gas separation performance in the next-generation MMMs can be realized by combining well-tuned hollow MOFs with fortified rigid interfaces.

Herein, we report a facile molecular soldering strategy to combine highly permeable PIMs (PIM-1) and highly porous MOFs via multifaceted polyphenol-mediation for engineering gas transport. Polyphenol soldering functions via two interlocked ways to overcome the permeability-selectivity trade-off in conventional MMMs: (1) the adhesive nature of polyphenol strikingly improves the polymer/MOF interfacial compatibility and chain rigidity; and (2) the polyphenols create a hollow architecture inside the MOF nanocrystal, reducing the transmembrane mass transfer resistance and compensating for the deleterious effect of the reduced chain mobility. Furthermore, the polyphenol-soldered MMMs could substantially suppress physical aging and resist plasticization. The efficiency of the polyphenol-soldering strategy is demonstrated on different polymers as a universal platform to prepare advanced MMMs with desirable enhanced performance.

## Results

### Materials synthesis and characterizations

**Fig. 1:**
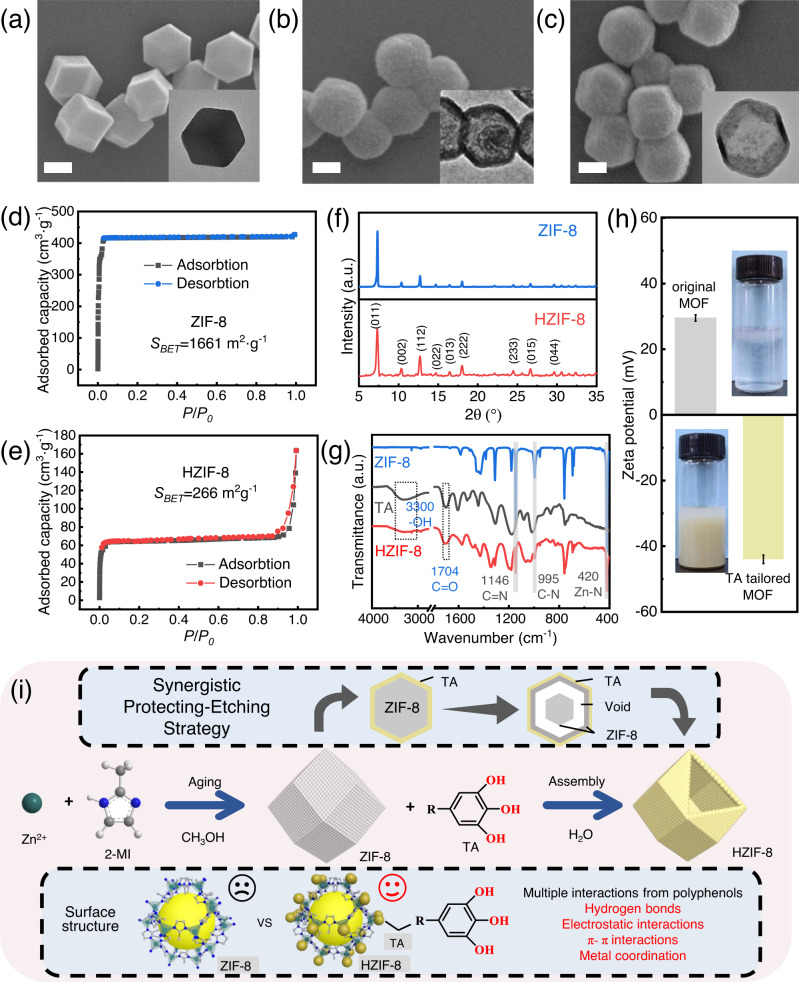
Effects of polyphenols on the physicochemical properties of MOF nanoparticles and the synthesis mechanism of hollow MOFs. **a**–**c** SEM images of ZIF-8 nanoparticles at different reaction times, **a** 0 min, **b** 2 min, and **c** 5 min. The inset picture is the corresponding TEM image (scale bars, 100 nm); **d**, **e** N_2_ adsorption-desorption isotherms of ZIF-8 (**d**) and HZIF-8 (**e**); **f** XRD patterns of ZIF-8 and HZIF-8; **g** FT-IR spectra of TA, ZIF-8, and HZIF-8; **h** Zeta potential of ZIF-8 and HZIF-8 in deionized water (the inset image shows the particles dispersed in water and left to stand for 1 h). Error bars represent the standard deviation of three independent measurements; **i** Schematic illustration of the synthetic process and mechanism for TA-tailored HZIF-8. The 2-MI represents 2-methylimidazole, the blue ball represents nitrogen atom, the gray ball represents carbon atom, and the white ball represents hydrogen atom.

Aqueous solutions of polyphenol tannic acid (TA) were used to tune the physicochemical structure of the synthesized MOF (zeolite imidazole framework: ZIF-8) crystals. As shown in the scanning electron microscopy (SEM) image, the original ZIF-8 particles are cubic crystals with approximate sizes of 200–300 nm (Fig. 1a). After TA modification, no significant changes are observed in the particle morphology, except that the surface becomes rougher (Fig. 1b, c). The transmission electron microscopy (TEM) images (insets of Fig. 1a–c) clearly show the gradual emergence of hollow ZIF-8 (HZIF-8) when the reaction time increases from 0 to 5 min.

The spatial configuration of HZIF-8 was confirmed via N_2_ isotherms and powder X-ray diffraction (XRD) (Fig. 1d–f). According to the International Union of Pure and Applied Chemistry (IUPAC) classification, ZIF-8 and HZIF-8 nanoparticles display classical type I and type II isotherm loops, respectively. The significant increase in HZIF-8 N_2_ adsorption at low pressure (*P/P*_*0*_ < 0.1) verifies its microporous structure^28^. The difference between ZIF-8 and HZIF-8 near the saturation vapor pressure (*P/P*_*0*_ > 0.9) exactly corresponds to the multilayer adsorption in the lumen of HZIF-8 as observed in the TEM images (insets of Fig. 1a–c). The Brunauer–Emmett–Teller (BET) surface area of ZIF-8 is 1661 m^2^ g^−1^, similar to the reported value^29^. In contrast, in HZIF-8, the BET surface area decreases to 266 m^2^ g^−1^ due to the eliminated inner mesoporous structure. The shell structure of HZIF-8 is also confirmed by the XRD patterns, wherein the diagnostic diffraction peaks are consistent with the synthesized ZIF-8 crystals, indicating that the topological configuration remains unchanged after TA modification.

The chemical properties of ZIF-8 and HZIF-8 nanoparticles were further characterized via Fourier transform infrared (FT-IR) spectroscopy (Fig. 1g). The characteristic peaks of both TA at 3300 cm^−1^ (-OH) and 1704 cm^−1^ (C = O) and ZIF-8 at 1146 cm^−1^(C = N), 995 cm^−1^ (C-N), and 420 cm^−1^(Zn-N) have been identified in the spectrum of HZIF-8. Given the diameter of TA molecules (>16 Å) and the pore size of ZIF-8 (3.4 Å), TA is assembled on the surface of ZIF-8 owing to the size distinction, and the X-ray photoelectron spectroscopy (XPS) results confirm this deduction (Supplementary Fig. 4 and Supplementary Table 2). The O *1s* peak in HZIF-8 increases distinctly, and the atomic concentration rises to 21.71% owing to the phenol hydroxyl group of TA. The thermal analysis result (Supplementary Fig. 5) shows that the TA mass ratio in HZIF-8 is approximately 30.9 wt.%. In addition, a change in the surface charge of the nanoparticles is indicated by the zeta potential (Fig. 1h). ZIF-8 particles are positively charged in aqueous solutions because of the unsaturated metal ions. In contrast, a large number of phenolic hydroxyl groups are on the surface of HZIF-8; the particles are negatively charged with higher hydrophilicity (Supplementary Fig. 6). Notably, these adhesive phenol hydroxyl groups can substantially facilitate the interaction between the polymer matrix and filler.

Figure 1i exhibits the synthesis mechanism of the polyphenol-modified MOFs and the corresponding structures via the synergistic protecting-etching method. First, the TA molecules coordinate with the exposed Zn^2+^ center in the ZIF-8 surface, stabilizing the overall crystal morphology and improving the surface hydrophilicity. Protons are then dissociated from TA and enter the crystal through the hydrophilic surface in the MOF. The original crystal structure is damaged due to the high pKa (7.75) of imidazole ligands^30^, which is confirmed by the variation in pH value (Supplementary Fig. 7) and the TEM images (Fig. 1a–c). Thus, TA acts not only as a surface modifier to increase the number of functional groups but also as an etching agent to create channels for high-speed gas transfer.

### Membrane characterizations

The synthesized ZIF-8 and HZIF-8 particles were dispersed in a highly permeable PIM-1 matrix to form MMMs via solution casting. The as-prepared membranes are denoted as Z-X-Y or H-X-Y, where Z and H represent the ZIF-8 and HZIF-8-based MMMs, respectively, and X and Y represent the relative mass ratio of PIM-1 to ZIF-8 from 5:0 to 5:5. Notably, Y represents the mass of ZIF-8 to maintain the same volume fractions of MOFs whether in PIM-1/ZIF-8 or PIM-1/HZIF-8 membranes because TA treatment does not change the number of MOF nanoparticles and the MOF volume. The maximum loading ratios are 5:3 for PIM-1/ZIF-8 and 5:5 for PIM-1/HZIF-8 due to the limitation of the mechanical strength of the resultant MMMs.

**Fig. 2:**
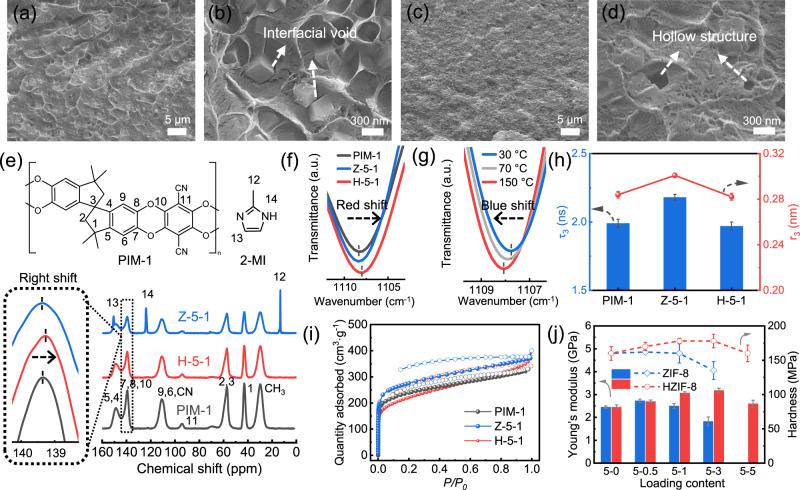
Membrane characterizations. **a**–**d** SEM images of cross-sections of Z-5-1 (**a**, **b**) and H-5-1 (**c**, **d**); **e** Solid ^13^C nuclear magnetic resonance (NMR) spectroscopy of PIM-1, Z-5-1, and H-5-1; **f** FT-IR spectra of aromatic ether group in PIM-1, Z-5-1, and H-5-1 membranes; **g** Temperature dependence of aromatic ether group absorption from 30 °C to 150 °C in H-5-1 membranes; **h** Positron annihilation lifetime spectroscopy (PALS) data of PIM-1, Z-5-1, and H-5-1 membranes. Error bars correspond to the standard deviations from three independent tests; **i** N_2_ isotherm at 77 K of PIM-1, Z-5-1, and H-5-1 membranes; **j** Young’s modulus and nanoindentation hardness of PIM-1/ZIF-8 and PIM-1/HZIF-8 MMMs at different loadings. Error bars correspond to the standard deviations, as obtained from six points.

As shown in the cross-sectional SEM images, the MOF crystals are uniformly dispersed in the PIM-1 matrix, while there is a visible difference in the polymer–filler interface between the Z-5-1 and H-5-1 membranes (Fig. 2a–d). Significant interface voids are observed in the PIM-1/ZIF-8 membrane, implying poor adhesion. In contrast, no voids are found at the interface of PIM-1/HZIF-8, suggesting that TA substantially modified the interfacial compatibility, which is critical for the high selectivity of PIM-1/HZIF-8. In addition, the hollow structure of HZIF-8 remains intact during the preparation process, which is conducive to the high-speed transfer of gas molecules. The surface morphology of the membranes is shown in Supplementary Fig. 8, wherein the surface of MMMs containing the TA-modified MOF is significantly smoother, also suggesting good adhesion of HZIF-8 to the PIM-1 matrix.

The chemical structure of the obtained MMMs was confirmed via solid ^13^C NMR spectroscopy (Fig. 2e). The chemical shift at 139.4 ppm corresponds to the aromatic ether groups, indicating successful synthesis of PIM-1. The ^13^C NMR spectrum of the H-5-1 membranes exhibits a slight rightward shift in the aromatic ether peak, demonstrating a change in the corresponding chemical environment which may be attributed to substantial hydrogen bonding between the hydroxyl groups in HZIF-8 and the PIM-1 chains. The FT-IR spectrum of the H-5-1 membrane (Fig. 2f) also exhibits a red shift for the aromatic ether groups at 1108 cm^−1^, whereas that of the Z-5-1 membrane exhibits no significant shift. The temperature dependence of the aromatic ether group absorption was also studied to qualitatively analyze the hydrogen bonds therein (Fig. 2g). Hydrogen bonds become weaker with increasing temperature^31^; therefore, the FT-IR spectrum of the H-5-1 membrane exhibits a clear blue shift with an increase in temperature from 30 to 150 °C. The NMR and FT-IR spectra unanimously prove the existence of hydrogen bonding between PIM-1 and HZIF-8, so that a significant difference in interfacial morphology has been detected in cross-sectional SEM images (Fig. 2a–d). The average chain spacing can be calculated from the XRD patterns according to Bragg’s equation (Supplementary Fig. 10) and reflects the characteristics of the polymer chain packing. Pristine PIM-1 displays a broad peak at 22.3°, corresponding to a d-spacing of 3.98 Å. Increasing the ZIF-8 content improves the chain spacing because ZIF-8 distributes the packing density^32^. However, increasing HZIF-8 content decreases the chain spacing. This suggests that HZIF-8 acts as a cross-linker and densely packs the surrounding polymer chains owing to its strong interaction with the PIM-1 chain, as confirmed by the ^13^C NMR and FT-IR spectra. In addition, XRD analysis of the MMMs verifies the integrity of ZIF-8 and HZIF-8 nanocrystals under arbitrary loading. Thermogravimetric analysis (TGA) reveals that introducing HZIF-8 has no noticeable effect on the thermal stability of PIM-1 at an operation temperature lower than 250 °C (Supplementary Fig. 11).

Positron annihilation lifetime spectroscopy (PALS, Fig. 2h and Supplementary Table 3) was used to examine the free volume elements in the membranes. Compared to pristine PIM-1, the free volume radii r_3_ and r_4_ of the Z-5-1 membrane increase from 2.84 and 4.52 Å to 3.01 and 4.61 Å, respectively, because of the loose chain packing and microporous structure of ZIF-8. In sharp contrast, the r_3_ value of H-5-1 membrane decreases from 2.84 to 2.82 Å, which is lower than that of the PIM-1 membrane. This unusual decrease implies that a substantial improvement in interfacial interaction reduces interchain spacing and densifies the overall packing, which is in agreement with the XRD analysis (Supplementary Fig. 10). In addition, the N_2_ isotherm (Fig. 2i and Supplementary Fig. 12) reflects a change in the pore structure of the MMMs. The median micropore sizes of pristine PIM-1, Z-5-1, and H-5-1 are 6.3, 7.0, and 5.6 Å, respectively. This trend is consistent with the PALS results, confirming the rearrangement of the PIM-1 chains, which is essential for improving the sieving ability of the membrane.

The mechanical properties of PIM-1/ZIF-8 and PIM-1/HZIF-8 were evaluated using a nanoindentation tester (Fig. 2j and Supplementary Fig. 13) to investigate the macro effects of polyphenol soldering on the polymer matrix. Usually, introducing inorganic nanoparticles in MMMs can improve the mechanical strength of the membranes; however, when interfacial defects (voids and aggregation) occur, the mechanical strength degrades rapidly. In this study, the mechanical properties of PIM-1/ZIF-8 membranes exhibited a decline at loading levels above 5:1, and a significant deterioration was observed at 5:3 with respect to pristine PIM-1. Comparatively, for PIM-1/HZIF-8 membranes, the mechanical properties are enhanced when the loading level ranges from 5:0 to 5:3 and start to degrade till 5:5. These two distinct trends imply that our soldering strategy helps achieve a uniform distribution of nanoparticles and optimize the polymer/MOF interfaces. Notably, this macroscopic mechanical property enhancement is also related to the microscopic rigidification of polymer chains because of polyphenol soldering. Moreover, these results verify the experimental findings that the PIM-1/ZIF-8 membranes are too fragile for gas separation as the loading level exceeds 5:3.

In Supplementary Fig. 14, we speculate the potential adhesions between PIM-1 and HZIF-8 to reveal the role of TA molecules at the MOF/polymer interface. The phenol–metal coordination firmly binds the TA molecules to the MOF surface. In addition, the high binding affinity of phenolic motifs at the MOF surface strengthens the polymer–filler connection, as verified by the detectable hydrogen bonds and potential π–π interactions among the benzene rings. These results explicitly suggest that polyphenols act as molecular solders to hold the matrix and filler together and strengthen the polymer/MOF interface.

### Gas transport performance

**Fig. 3:**
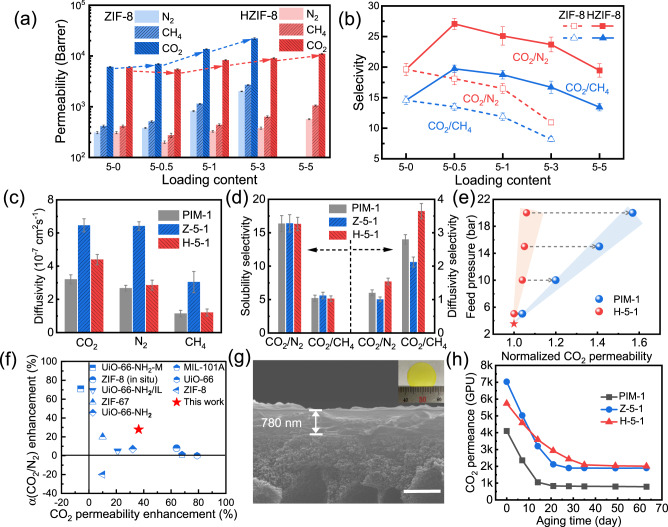
Gas transport performance. **a**, **b** Gas permeability (**a**) and ideal selectivity (**b**) in MMMs as a function of filler content at 35 °C and 3.5 bar. Error bars represent the standard deviations obtained from three membranes; **c**, **d** Diffusivity (**c**) of CO_2_, N_2_, and CH_4_, and sorption and diffusion selectivity (**d**) of CO_2_/N_2_, CO_2_/CH_4_ in PIM-1, Z-5-1, and H-5-1. Error bars represent the standard deviations obtained from three membranes; **e** Normalized CO_2_ permeability of PIM-1 and H-5-1 membranes with CO_2_ feed pressure increasing from 3.5 to 20 bar; **f** Comparison of the CO_2_ permeability versus CO_2_/N_2_ selectivity improvement for PIM-1/MOF MMMs in the literature with the optimal result in this study. The blue dots represent the data from references, as listed in Supplementary Table 7, and the red star represents the H-5-1 membrane. Source data are provided as a Source Data file; **g** Cross-sectional SEM image of the H-5-1 thin-film membrane on the surface of PI support. The inserted picture is the corresponding macro digital photo (Scale bar: 1 μm); **h** Ageing behavior of PIM-1, Z-5-1, and H-5-1 thin-film membranes. The samples are directly exposed to the atmosphere during the test interval.

The pure gas-transport properties were studied using a constant volume-variable pressure method. For the PIM-1/ZIF-8 membranes, the permeability to all gases increases dramatically when more ZIF-8 is loaded, while the ideal selectivity continuously decreases (Fig. 3a, b). The CO_2_ permeability rises from 6065 to 22046 Barrer when the ZIF-8 ratio is increased from 5:0 to 5:3, and the CO_2_/N_2_ and CO_2_/CH_4_ selectivity decreases from 19.6 to 11.0 and 14.6 to 8.2, respectively. This opposite trend is typical in conventional MMMs involving nonselective diffusion, as observed in the cross-sectional SEM image in Fig. 2b.

Conversely, for the PIM-1/HZIF-8 membranes, the CO_2_ permeability remains almost unchanged, and a substantial increase (from 19.6 to 27.1 for CO_2_/N_2_ and from 14.6 to 19.7 for CO_2_/CH_4_) is observed at a low loading of 5:0.5. As more HZIF-8 is loaded, the gas permeability increases and the selectivity starts to decrease. The optimum point appears in H-5-1 with a CO_2_ permeability of 8268 Barrer and CO_2_/N_2_ and CO_2_/CH_4_ selectivity of 25.1 and 18.7, respectively. Even at the highest loading level (H-5-5), the selectivity (19.5 for CO_2_/N_2_ and 13.4 for CO_2_/CH_4_) is similar to that of the pristine PIM-1 membrane. Moreover, the mixed gas separations for pristine PIM-1 and H-5-1 membranes are shown in Supplementary Table 5. Owing to competitive adsorption^33^, the permeability and selectivity of both membranes are reduced, but the H-5-1 membrane still presents a significant superiority.

To gain insight into the gas-transport mechanism in this polyphenol-soldered MMM, a solution-diffusion model was employed to calculate the sorption and diffusion coefficients via the time-lag method (Fig. 3c, d, and Supplementary Fig. 15). Minor improvements in gas solubility are detected with the incorporation of ZIF-8 and HZIF-8, reflecting the moderate sorption capacity between the MOF crystals and microporous PIM-1. Unlike the slightly improved sorption coefficient, the change in diffusivity is pronounced. In Z-5-0.5, the diffusivity remains relatively stable. However, as the ZIF-8 content continues to increase, the diffusion coefficient appears to increase exponentially, accompanied by a significant decrease in diffusion selectivity (from 2.80 to 1.51 for CO_2_/CH_4_ and from 1.21 to 0.77 for CO_2_/N_2_). This unexpected variation suggests the presence of nonselective voids between the PIM-1 and ZIF-8 particles, which is consistent with the cross-sectional SEM images and decreased mechanical properties. In sharp contrast, at a low loading of HZIF-8 (H-5-0.5), a slight decrease in CO_2_ diffusivity and a significant increase in diffusivity selectivity (from 2.80 to 3.80 for CO_2_/CH_4_ and from 1.21 to 1.60 for CO_2_/N_2_) are observed, which imply good adhesion at the polymer–filler interface. The variations in the diffusion coefficient and selectivity are presumably due to the rearrangement and stiffening of the polymer chains, as proved by the PALS and XRD results (Fig. 2h and Supplementary Fig. 10). At higher HZIF-8 loadings (H-5-1 and H-5-3), the fillers may be connected and hence facilitate gas transport. This in turn enhances gas diffusivity and reduces diffusivity selectivity; however, it is still higher than that of PIM-1 and PIM-1/ZIF-8 membranes (Supplementary Fig. 15f). When the loading level reaches 5:5, HZIF-8 inevitably agglomerates within the PIM-1 matrix, leading to the substantial loss of diffusivity selectivity as evidenced by a significant deterioration of mechanical strength (Fig. 2j).

The separation performance of the PIM-1/HZIF-8 MMMs and pristine PIM-1 membrane was compared under a critical high feed pressure. When polymer membranes are used for high-pressure CO_2_ separation, the solution of CO_2_ in the membrane usually increases the mobility of the polymer chains, decreasing the separation selectivity (known as “plasticization”)^34^. In Fig. 3e, the PIM-1 membrane exhibits significant plasticization during continuous pressure boosting tests, and the CO_2_ permeability at 20 bar is 1.57 times higher than that at 3.5 bar. In contrast, the increase in CO_2_ permeability is only 6% in the PIM-1/HZIF-8 membrane, which is significantly lower than that in the PIM-1 membrane. The impressive suppression of plasticization in the PIM-1/HZIF-8 membrane demonstrates that the substantial interaction between TA-tailored MOFs and the PIM-1 matrix improves the rigidity of the polymer chains.

Figure 3f and Supplementary Table 7 present a comparison of our data with those reported in recent PIM-1/MOF studies. The present study aims to fabricate MMMs with simultaneously enhanced permeability and selectivity, whereas most reported PIM-1/MOF MMMs can only achieve a satisfactory increase in CO_2_ permeability. However, selectivity is more pertinent for PIMs, which are known for their high gas permeability^35^. Although the incorporation of UiO-66-NH_2_-M can increase CO_2_/N_2_ selectivity by 71%, it has a significant negative effect on gas permeability owing to the rigidification of the polymer^36^. Encouragingly, our synthesized PIM-1/HZIF-8 membrane (H-5-1) can achieve a simultaneous increase in both permeability (up to 36%) and selectivity (up to 28%). As shown in Fig. 3f, our soldering strategy illustrates overwhelming superiority to the other works by overcoming the trade-off limit between permeability and selectivity.

Physical aging is the relaxation of polymer chains to a denser packing state after a period, which is particularly common in glassy polymers^37^. PIM-1, with a nonequilibrium state due to inefficient packing, is well known for the loss of membrane performance over time^38^. Therefore, the aging behavior of thin-film composite membranes was examined here for practical gas separation purposes. As shown in the cross-sectional SEM images in Fig. 3g and Supplementary Fig. 17, the thickness of the thin film is approximately 800 nm. In the 63-day aging tests (Fig. 3h), the H-5-1 membrane exhibits more favorable aging performance compared with pristine PIM-1 and Z-5-1, which can be revealed from three aspects. (1) In the first week, the CO_2_ permeance of the pristine PIM-1 and Z-5-1 membranes decrease by 42.5% and 28.5%, respectively, whereas that for H-5-1 is 20.2%. (2) It takes approximately 8 and 13 days for CO_2_ permeance to decrease to 50% for the PIM-1 and Z-5-1 membranes, whereas for the H-5-1 membrane, 23 days are required. (3) By the ninth week of the aging test, the permeance of pristine PIM-1 and Z-5-1 membranes drop by 81% and 73%, respectively, while the H-5-1 membrane drops by 60%. Thus, through multiple interactions arising from polyphenol soldering, the PIM-1 chains are rigidified, mitigating the molecular rearrangement and lattice contractions within the PIM-1 matrix.

**Fig. 4:**
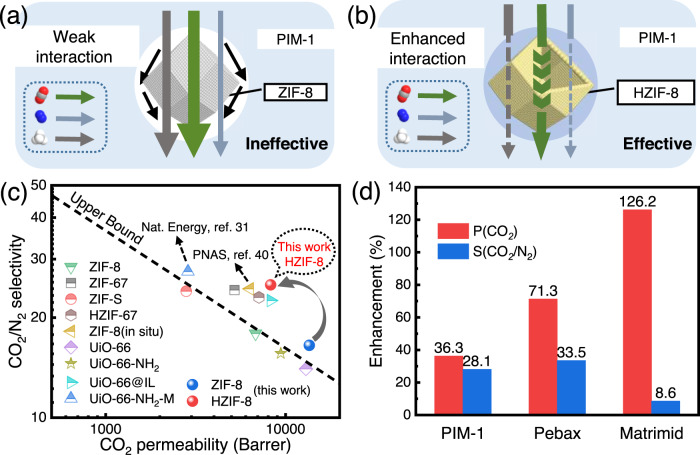
Mechanism and superiority of the polyphenol-soldering strategy. **a**, **b** Gas-transport mechanism in PIM-1/ZIF-8 (**a**) and PIM-1/HZIF-8 (**b**) MMMs. The gas molecules in the dotted box represent CO_2_, N_2_, and CH_4_ from top to bottom; **c** Comparison of CO_2_/N_2_ separation performance obtained in this work with other reported PIM-1/MOF MMMs. Detailed comparison data are available in Supplementary Table 8. Source data are provided as a Source Data file; **d** Gas separation performance of HZIF-8 within different polymers. P(CO_2_) and S(CO_2_/N_2_) represent the CO_2_ permeability and CO_2_/N_2_ selectivity, respectively. The values on each column stand for the specific enhancement of corresponding membranes.

The possible gas-transport mechanisms of the conventional PIM-1/ZIF-8 membrane and our multifaceted polyphenol-soldered PIM-1/HZIF-8 membranes are presented in Fig. 4a, b, respectively. Due to the weak interaction between the MOF and the polymer matrix, nonselective interfacial voids are easily generated and deteriorate the separation efficiency^39,40^, especially in the glassy PIM-1 prepared via conventional fabrication methods. Comparatively, via multifunctional polyphenols, HZIF-8 acts as a noncovalent cross-linking node to join the adjacent polymer chains. Thus, the integral chain migration is restricted, improving the selectivity of PIM-1/HZIF-8 MMMs. In addition, polyphenols create an inner cavity in ZIF-8 and reduce the overall diffusion resistance. Therefore, our polyphenol-soldering strategy based on modified MOFs has the potential to drive concurrent advances in the permeability and selectivity of MMMs.

Figure 4c presents a comparison of the separation performance of PIM-1/ZIF-8 and PIM-1/HZIF-8 using the polymeric upper-bound plot^41^. As expected, the PIM-1/HZIF-8 membrane easily surpasses the upper-bound line and demonstrates significant superiority to various reported PIM-1/MOF MMMs. The polyphenol-soldering strategy was also applied to two attractive membrane separation materials, i.e., rubbery polyethylene oxide (PEO, Pebax) and glassy polyimide (Matrimid). Figure 4d shows the same synchronous enhancement in both CO_2_ permeability and CO_2_/N_2_ selectivity among three different polymers. These results demonstrate that the polyphenol-based soldering approach provides a general toolbox for enhancing the separation properties of MMMs.

## Discussion

In summary, based on tailored polymer chains, well-designed hollow MOF structures, and defect-free interfaces, we demonstrated an attractive mixed-matrix approach via facile polyphenol soldering to achieve rational matching of polymers and fillers. The adhesive nature of polyphenols promotes complex interactions with PIM-1 chains, thus increasing the chain stiffness and selectivity. Moreover, the assembly of polyphenols on the surface endows the MOF with a unique hollow structure, reducing the mass transfer resistance. With the multifunctional polyphenols, both gas permeability and selectivity could increase synchronously in contrast to the trade-off of permeability and selectivity in conventional MMMs. The well-adhered MOF particles within the polymer matrix improve the physical aging and plasticization of PIM-1 and maintain the long-term stability of the thin-film composite membranes. Our proposed interfacial soldering approach perfectly combines two well-known materials while preserving their inherent advantages. This approach provides an inspiring solution to the long-standing issue of unsatisfactory matching between the fillers and matrix, paving the way for the preparation of advanced MMMs with desirable performance for various applications beyond carbon capture.

## Methods

### Material

5,5’,6,6’-Tetrahydroxy-3,3,3’,3’-tetramethyl-1,1’-spirobisindane (TTSBI, 97%, Alfa Aesar) was purified by dissolving in methanol and re-precipitating from dichloromethane. 2,3,5,6-Tetrafluoroterephthalonitrile (TFTPN, 99%, Sigma-Aldrich) was purified via sublimation. Zinc acetate dihydrate (Zn(CH_3_COO)_2_·2H_2_O), dimethylimidazole (Hmim), anhydrous potassium carbonate (K_2_CO_3_), and tannic acid (TA) were supplied by Aladdin with analytical grade. Dimethylformamide (DMF), chloroform (CHCl_3_), cyclohexane, methanol, and ethanol were purchased from Macklin. Deionized water was used in this work.

### Synthesis of nanoparticles

ZIF-8: Zn(CH_3_COO)_2_·2H_2_O (175 mg) and Hmim (263 mg) were dissolved in 20 ml methanol, respectively. After complete dissolution, the two solutions were mixed, stirred for 5 min, and then transferred to a thermotank at 30 °C for 24 h. Finally, the white products were centrifuged and washed with methanol. The ZIF-8 powder was dried and activated at 100 °C overnight before usage.

HZIF-8: ZIF-8 (10 mg) was dispersed in 5 ml of deionized water. Then, 5 ml TA solution (10 g/L) was added to the ZIF-8 suspension, followed by standing for 5 min. The products were centrifuged and washed with water.

### Synthesis of PIM-1

TTSBI (3.41 g) and TFTPN (2.01 g) were mixed in DMF (30 ml), followed by the addition of cyclohexane (150 ml) and K_2_CO_3_ (4.0 g). The reaction was conducted at 60 °C for 72 h with the protection of N_2_. The product was dissolved in CHCl_3_ and precipitated by methanol for purification. The obtained yellow powder was dried at 80 °C for 24 h.

### Fabrication of mixed-matrix membrane

PIM-1-based MMMs were fabricated by a priming process. PIM-1 (0.1 g) was dissolved in CHCl_3_ (9.9 g) to obtain a 1 wt% solution. A certain amount of nanoparticles based on the loading ratio was dispersed in CHCl_3_ and sonicated for 1 h, followed by addition into 1 ml of PIM-1 solution. After 8 h of stirring, the remaining PIM-1 solution was dropped into the above dispersion and stirred for another 8 h. Finally, the casting solution was poured into a homemade mold, and the evaporation process was controlled for no less than 2 days. The obtained membranes were activated in methanol for 24 h before the test. Thin-film composite membranes were obtained by spin-coating a 1 wt% PIM-1 solution on the surface of polyimide (PI) substrate cast by a nonsolvent phase inversion method.

### Characterization

The chemical structure of the synthesized powders and membranes was examined by FTIR (Thermo Scientific Nicolet is50, 4000–400 cm^−1^) and XPS (Shimadzu AXIS Ultra DLD, Al-Kα). The zeta potential of the nanoparticles was determined using a MARLVEN ZS-90. The crystalline structures were characterized using a Bruker D8 ADVANCE X-ray diffractometer and PANalytical X’Pert PRO (Cu-Kα, 40 mA, 40 kV). A transmission electron microscope (Tecnai T20) was used to characterize the hollow structure of synthesized MOFs. The morphology of MOFs and cross-section of membranes was observed by FESEM (ZEISS Sigma 300). The N_2_ absorption isotherms of MOF particles and membranes were conducted by BSD-660M A6B3M (Beishide Instrument Technology (Beijing) Co., Ltd) at 77 K. The sample was degassed at 100 °C for 450 min before the absorption test. Thermogravimetric analysis was conducted by PerkinEImer TGA400 from room temperature to 800 °C in a ratio of 10 °C/min under N_2_ or air atmosphere. Differential scanning calorimetry (DSC) analysis was controlled from −90 °C to 250 °C with a heating rate of 10 °C/min. In-situ FT-IR tests were performed on a BRUKER VERTEX 80 from 30 to 150 °C provided by eceshi (www.eceshi.com). The microphase structure was obtained by small-angle X-ray scattering (SAXS) via Saxesess mc2 (Anton Paar, Germany) with Kα radiation from 0.08 to 5° at 30 °C. The free volume size and free volume distribution of the membranes were detected by positron annihilation lifetime spectroscopy (PALS) with a PATFIT program. ^22^Na isotope was employed as positron and g-ray (1274 KeV) sources. The membrane samples were cut into pieces of 1 × 1 cm, and the thickness of each sample was controlled at approximately 1 cm.

## Supplementary information

Supplementary Information

## Data Availability

Source data are provided with this paper. The data used in this study are presented in the text, Supplementary Information, and Source Data. Additional data and information are available from the corresponding author upon request.
