# Supplementary material for: Boosting membrane carbon capture via multifaceted polyphenol-mediated soldering
Source: Nat Commun. 2023 Mar 27;14:1697. doi: 10.1038/s41467-023-37479-9 (PMC10043006; doi:10.1038/s41467-023-37479-9)
Supplement: Supplementary file 1 — Supplementary Information [file 41467_2023_37479_MOESM1_ESM.pdf]

Supplementary Information

Boosting Membrane Carbon Capture via Multifaceted Polyphenol-mediated Soldering

Zhu et al.

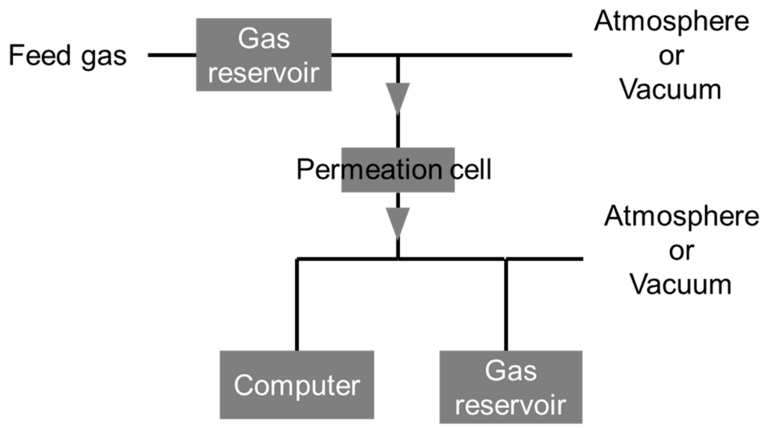

Supplementary Figure 1. Schematic diagram of pure gas permeation test.

Supplementary Note 1: The pure gas permeation equipment is based on a constant volume -variable pressure method from Suzhou Faith and Hope Membrane Technology Co. Ltd. The feed gas pressure varied from 3.5 to 20 bar and the permeate side kept vacuum. The operating temperature ranged from 35 to 55 °C. The permeation data was obtained from the average of three membrane samples to make sure reproducibility. Pure gas permeability was tested in the sequence H_2, N_2, CH_4, and CO_2.

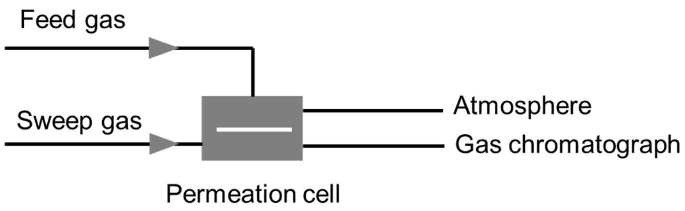

Supplementary Figure 2. Schematic diagram of mixed gas permeation test.

Supplementary Note 2: Binary gas CO_2/N_2 (10/90vol%) and CO_2/CH_4 (50/50vol%) permeation experiments were conducted based on a constant pressure/variable volume method. Helium is sweep gas. The feed pressure was kept at 2 bar and the test temperature was at 35 °C. The flow rates of feed gas and sweep gas were controlled by two mass flowmeters at 100 ml/min and 40 ml/min, respectively. The flow rate of permeation side was obtained by a flow meter and the composition was determined by a gas chromatograph. Each sample was tested for at last three times.

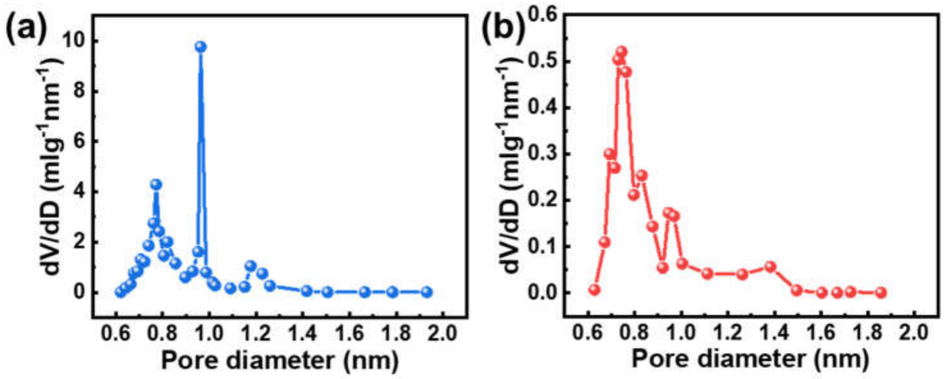

Supplementary Figure 3. Pore size distribution of ZIF-8 (a) and HZIF-8(b) from the H-K model.

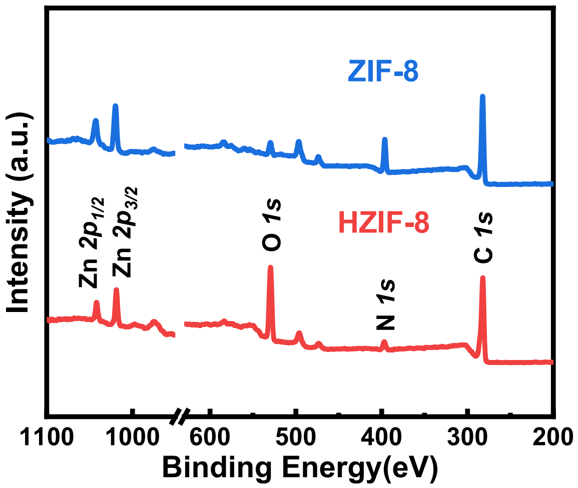

Supplementary Figure 4. XPS spectra of ZIF-8 and HZIF-8 nanoparticles.

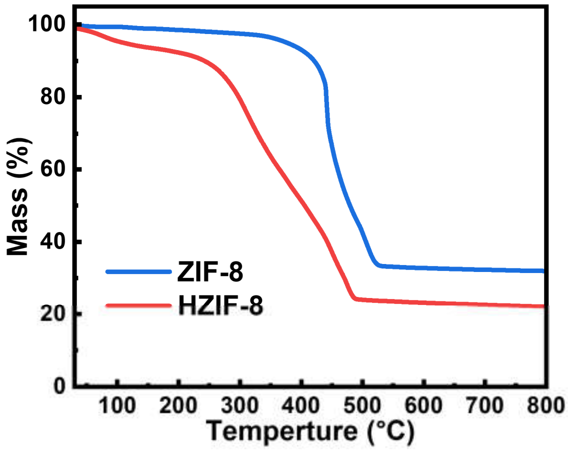

Supplementary Figure 5. TGA curves of ZIF-8 and HZIF-8 under air atmosphere.

Supplementary Note 3: The TA loading is evaluated by thermo gravimetric analysis in air. After 800 °C treatment, the remaining material is completely converted to zinc oxide. So the TA mass ratio in HZIF-8 (α) can be calculated according to following equal.

$$\alpha = 1 - \frac{R_{HZIF-8}}{R_{ZIF-8}}$$

where R_{HZIF-8} and R_{ZIF-8} is the residual weight of HZIF-8 and ZIF-8, separately.

According to Supplementary Figure 5, the values for R_{HZIF-8} and R_{ZIF-8} are 22.1 wt% and 32.1 wt%. So, the TA mass ratio in HZIF-8 is about 30.9 wt%.

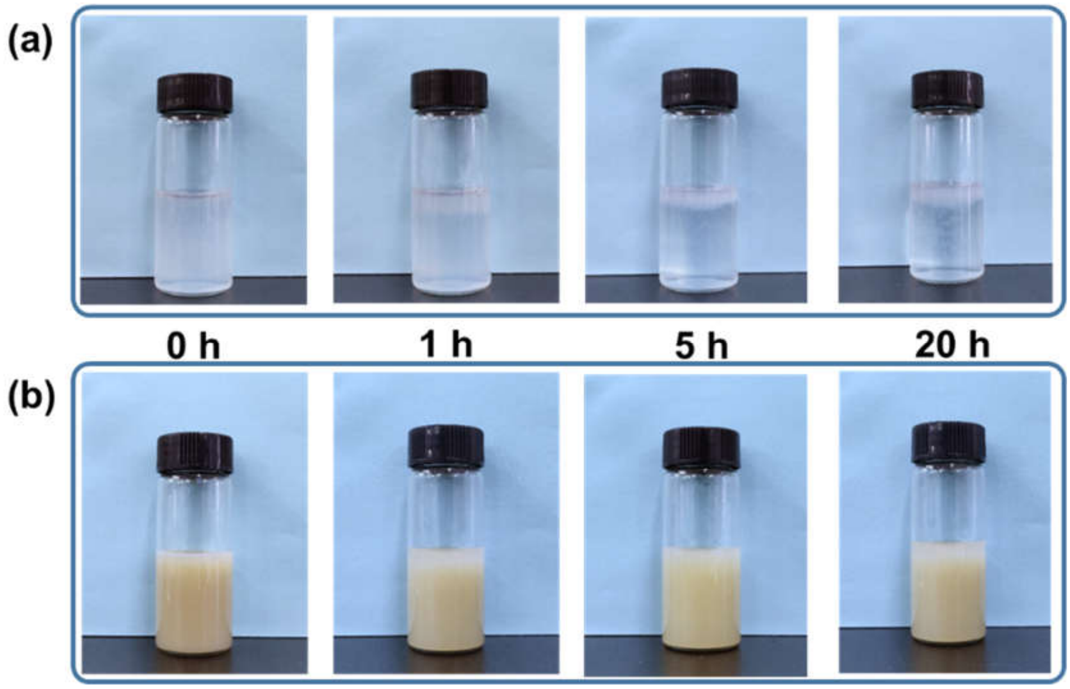

Supplementary Figure 6. Photographs of ZIF-8 (a) and HZIF-8 (b) with equal volume fraction dispersed in water.

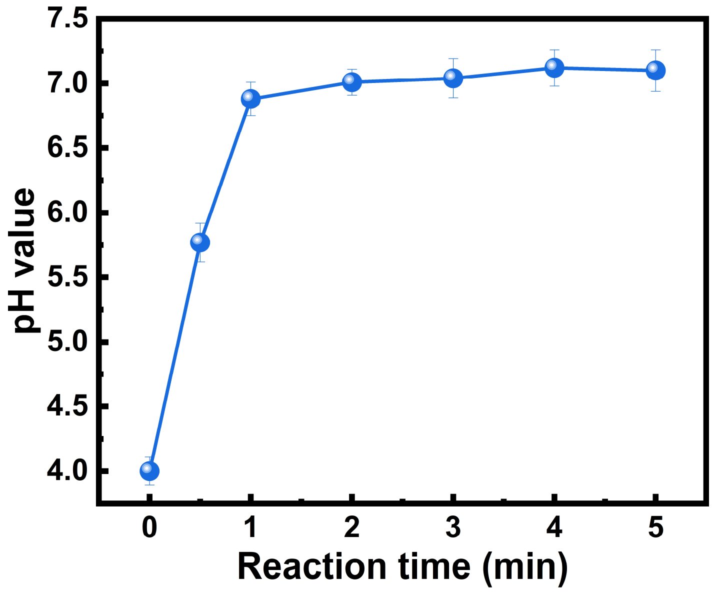

Supplementary Figure 7. pH value of the ZIF-8 particles in TA solution as a function of time.

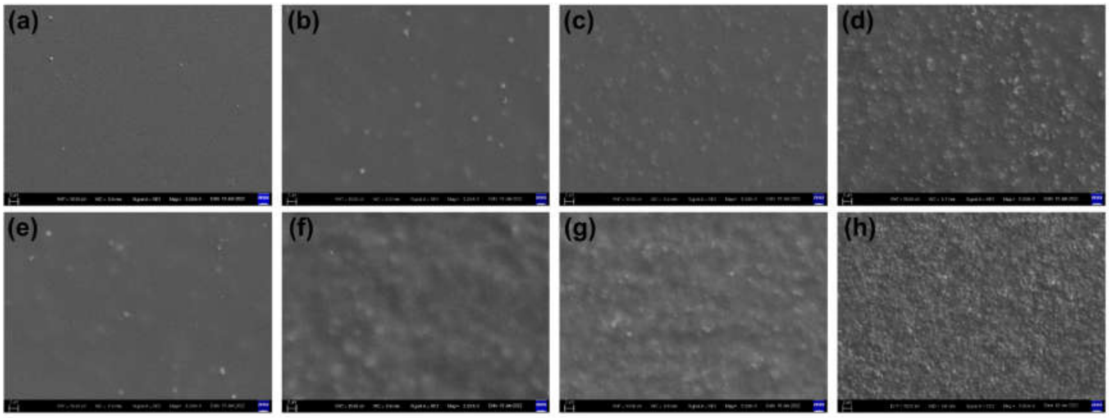

Supplementary Figure 8. Surface SEM images of (a) PIM-1, (b) Z-5-0.5, (c) Z-5-1, (d) Z-5-3, (e) H-5-0.5, (f) H-5-1, (g) H-5-3, (h) H-5-5

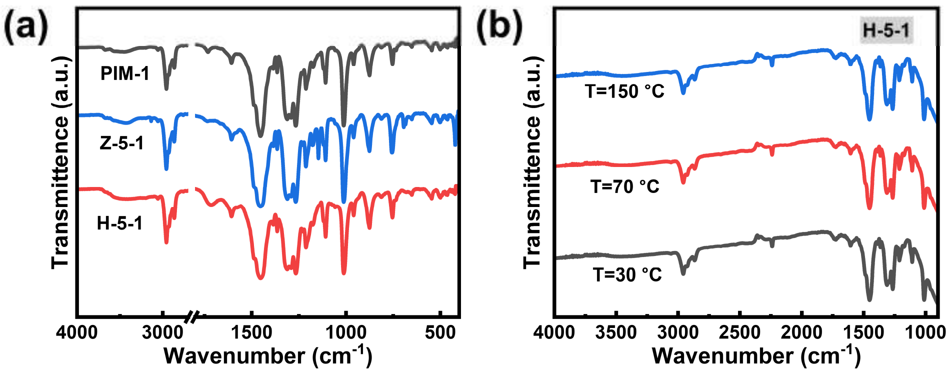

Supplementary Figure 9. (a) FTIR spectra of PIM-1, Z-5-1, and S-5-1 membranes; (b) In situ FT-IR spectra of H-5-1 membranes from 30 °C to 150 °C.

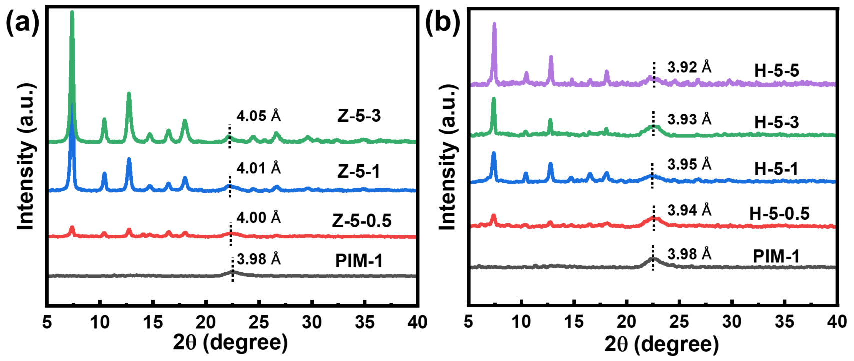

Supplementary Figure 10. XRD spectra of PIM-1/ZIF-8 (a) and PIM-1/HZIF-8 (b) membranes.

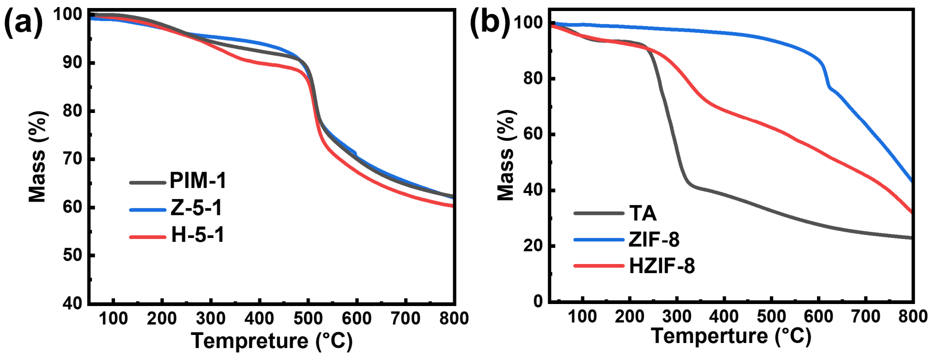

Supplementary Figure 11. TGA curve of (a) PIM-1, Z-5-1, and H-5-1 and (b) TA, ZIF-8, and HZIF-8 under N_2 atmosphere.

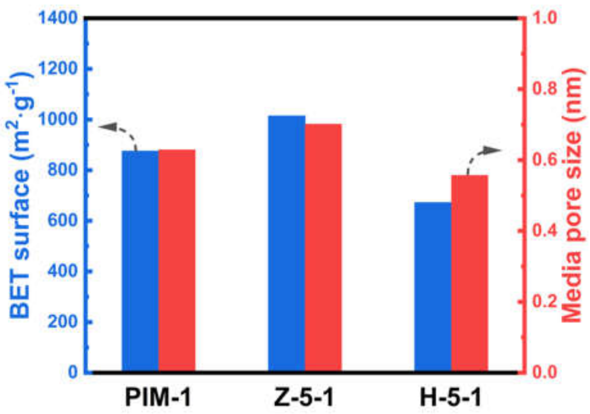

Supplementary Figure 12. BET surface area and media pore size of PIM-1, Z-5-1, and H-5-1 membranes.

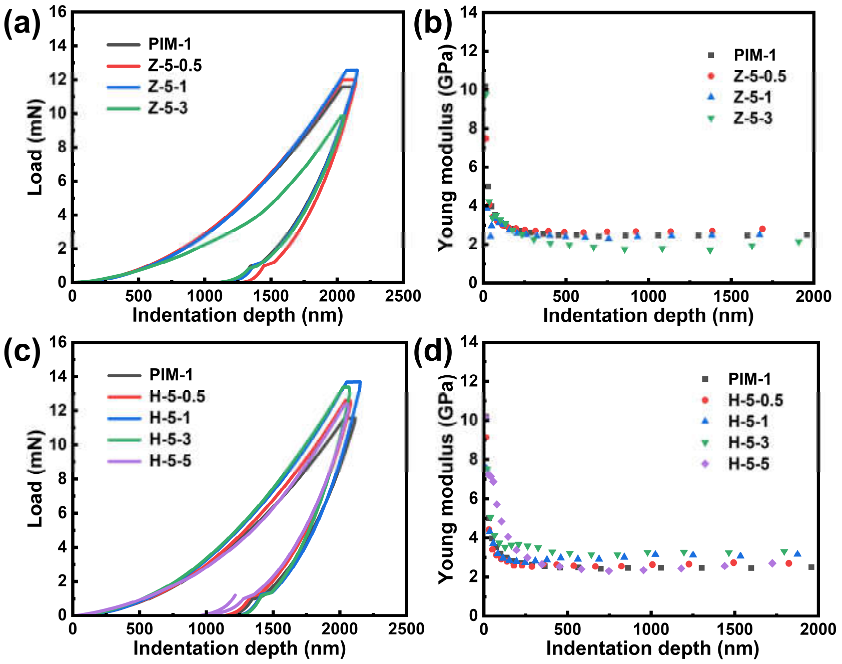

Supplementary Figure 13. Mechanical properties. Load of PIM-1/ZIF-8 (a) and PIM-1/HZIF-8 (c) as a function of indentation depth, Young modulus of PIM-1/ZIF-8 (b) and PIM-1/HZIF-8 (d) as a function of indentation depth.

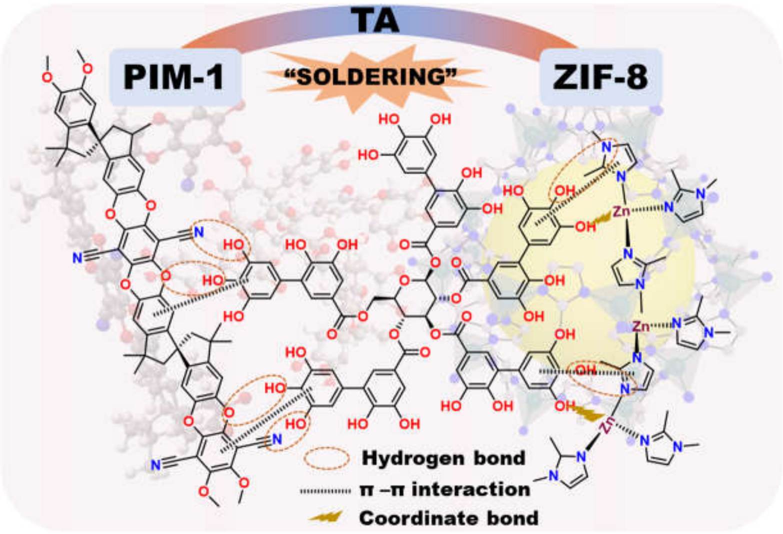

Supplementary Figure 14. Potential adhesions among PIM-1 chains, TA molecules, and ZIF-8.

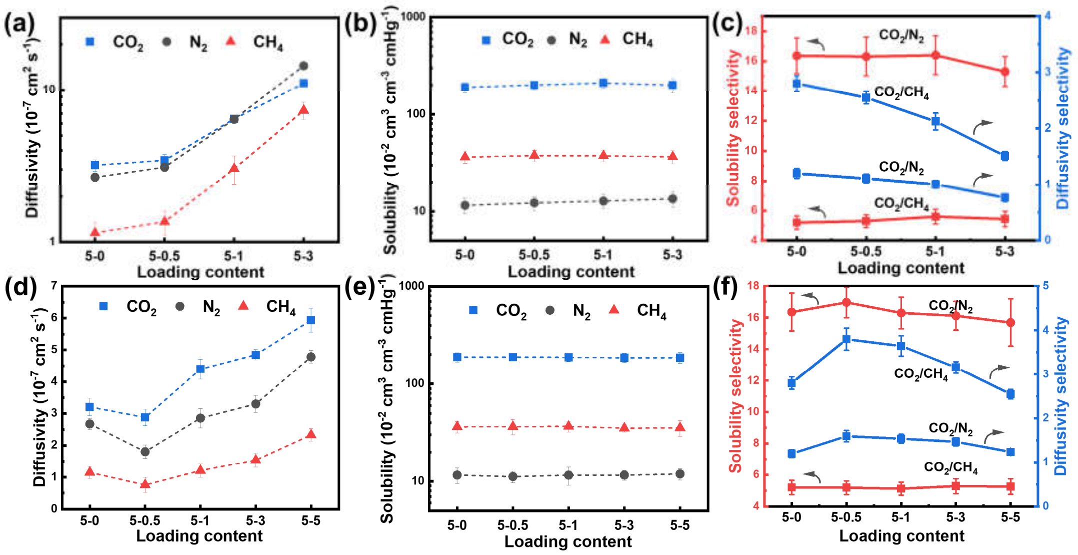

Supplementary Figure 15. Parameter of diffusion coefficients (a), solubility (b), and solubility and diffusivity selectivity (c) in PIM-1/ZIF-8 membranes. Parameter of diffusion coefficients (d), solubility (e), and solubility and diffusivity selectivity(f) in PIM-1/ HZIF-8 membranes. Error bars in all figures represent standard deviation.

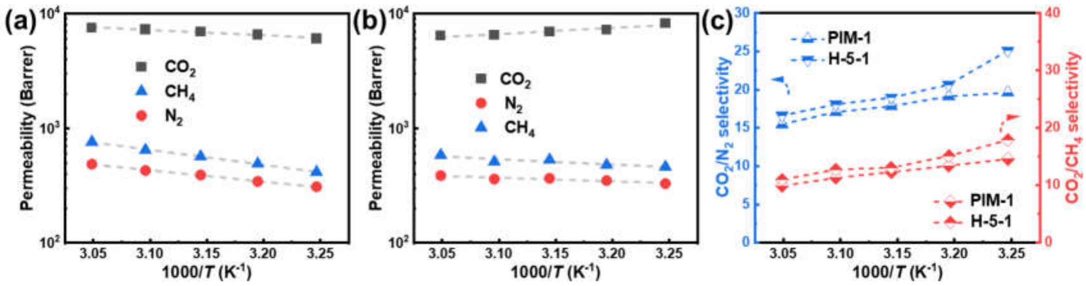

Supplementary Figure 16. Effect of temperature on gas permeability of PIM-1 (a), H-5-1 (b), and gas selectivity (c) at 3.5 bar.

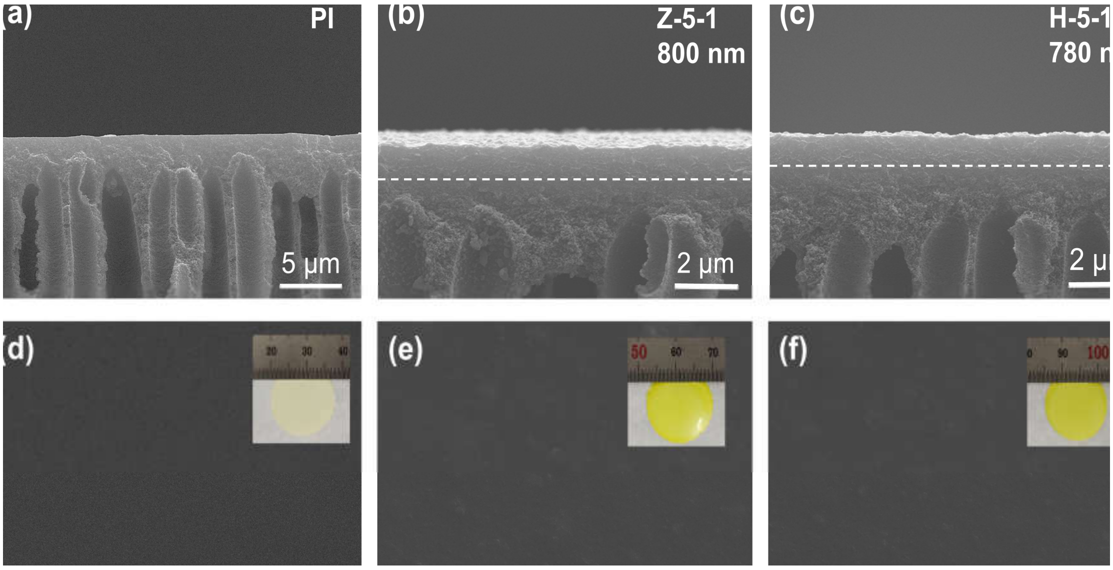

Supplementary Figure 17. SEM images of thin-film composite membrane. cross-sectional SEM image of (a) PI substrate; (b) Z-5-1; (c) H-5-1. Surface SEM image of (d) PI substrate, (e) Z-5-1; (f) H-5-1. The inserted photos in d,e,f represents the digital pictures of obtained membranes.

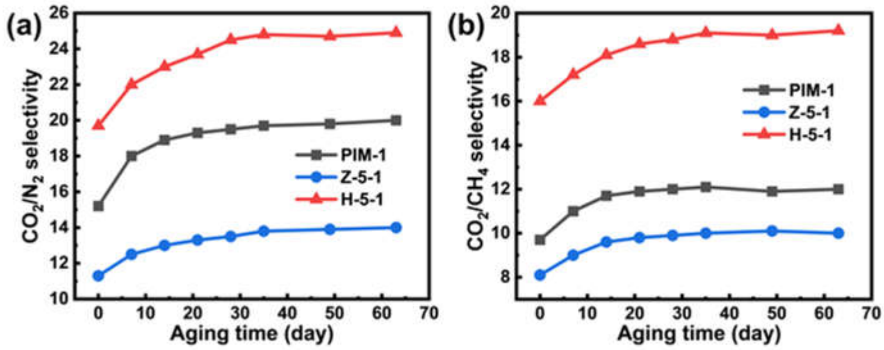

Supplementary Figure 18. CO_2/N_2 (a) and CO_2/CH_4 (b) selectivity of thin-film composite membranes as a function of aging time.

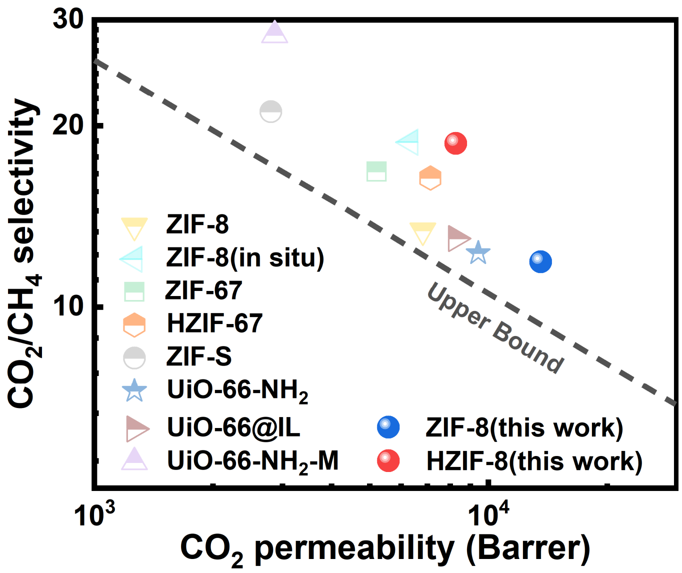

Supplementary Figure 19. Comparison of CO_2/CH_4 separation performance with Robeson upper bound.

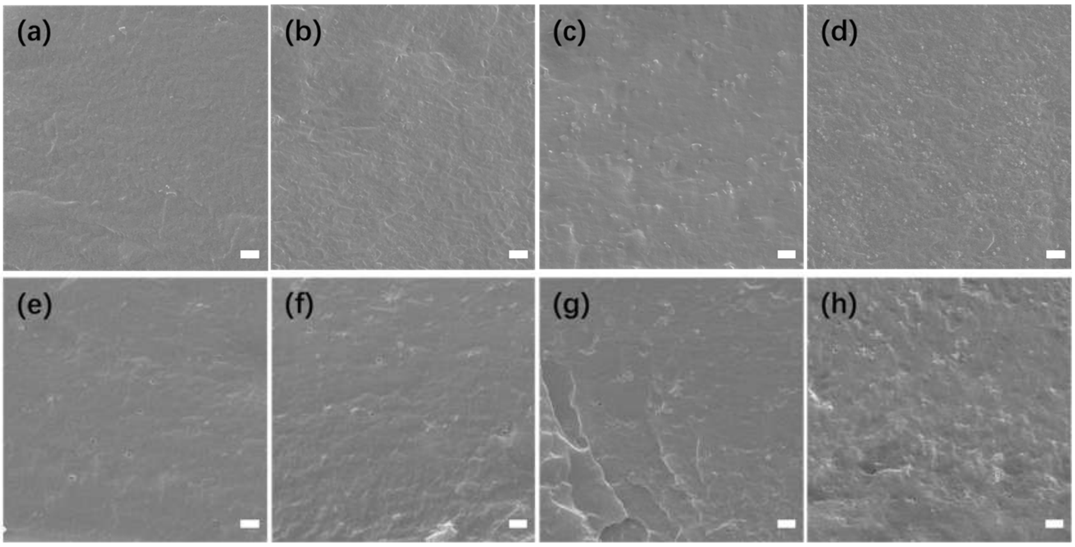

Supplementary Figure 20. Cross-section of Pebax based mixed matrix membranes. (a-d) ZIF-8 containing membranes with mass ratio 1, 3, 5 and 10%; (e-h) HZIF-8 containing membranes with the same volume fraction with Pebax/ZIF-8 membrane.

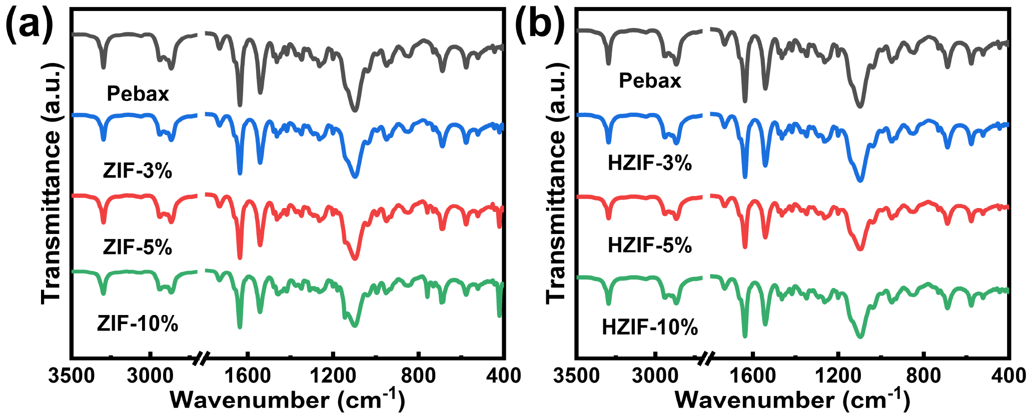

Supplementary Figure 21. ATR-FTIR spectra of Pebax/ZIF-8 (a) and Pebax/HZIF-8 (b) membranes

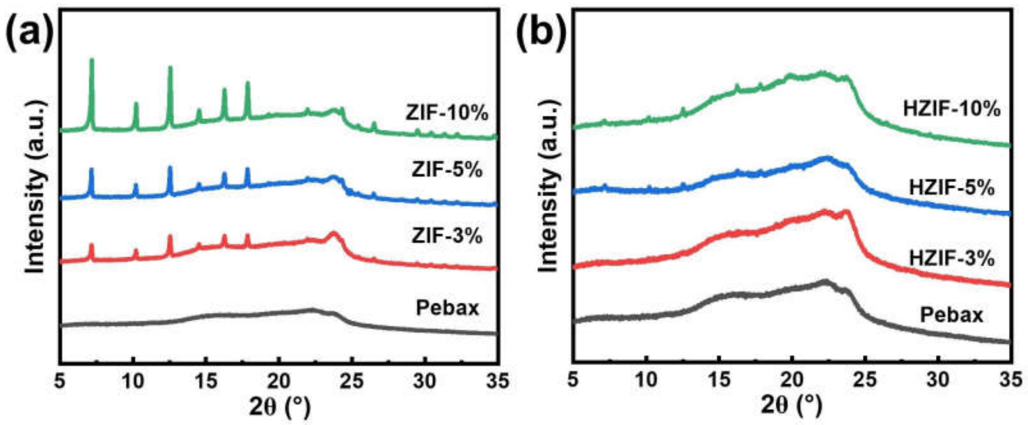

Supplementary Figure 22. XRD spectra of Pebax/ZIF-8 and Pebax/HZIF-8 membranes.

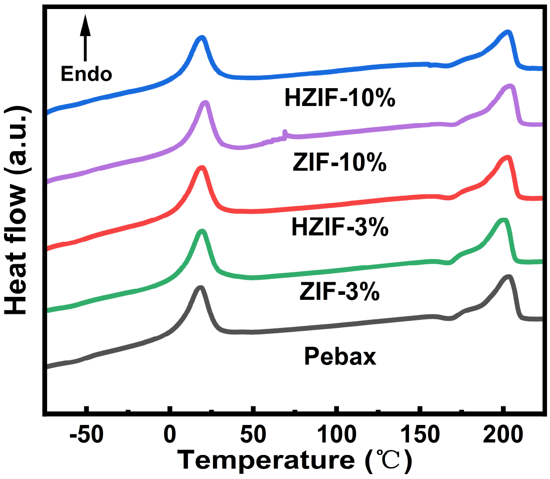

Supplementary Figure 23. DSC curve of Pebax/ZIF-8 and Pebax/HZIF-8 membranes.

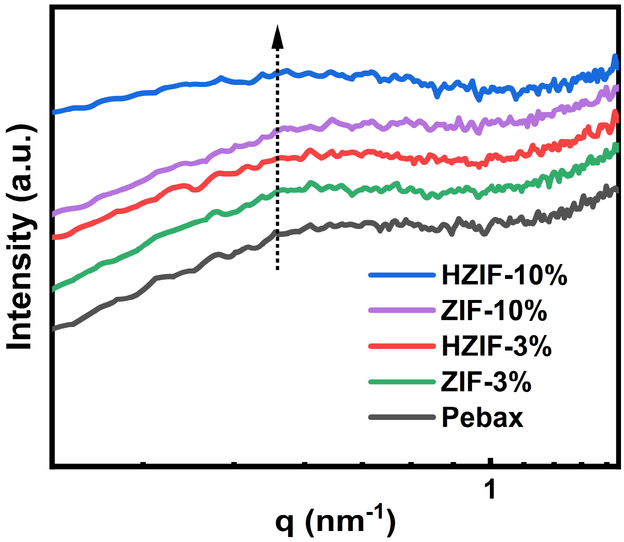

Supplementary Figure 24. SAXS curve of Pebax/ZIF-8 and Pebax/HZIF-8 membranes

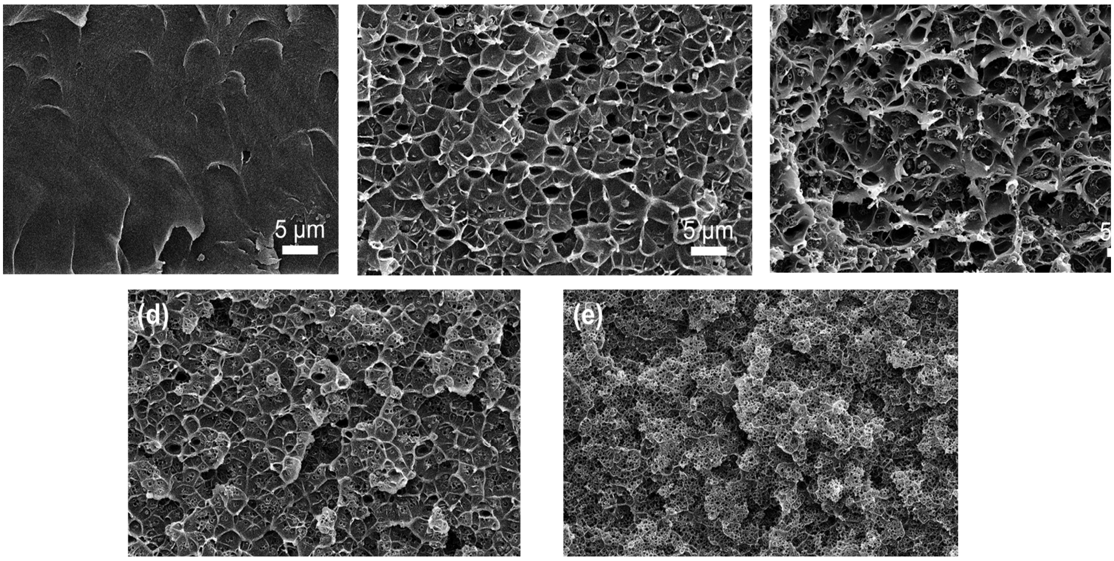

Supplementary Figure 25. Cross-section SEM images of (a) M-0, (b) M-5, (c) M-10, (d) M-20, and (e) M-30.

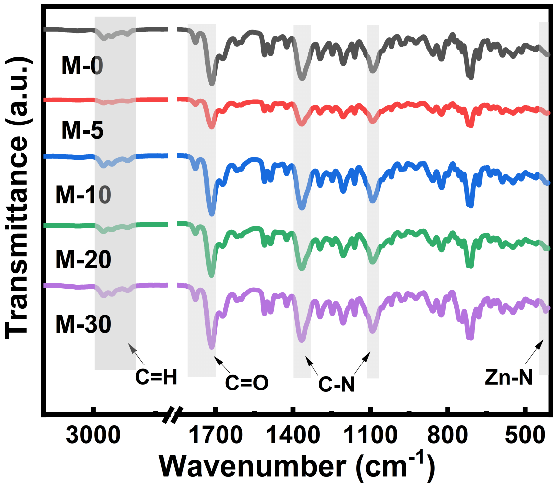

Supplementary Figure 26. ATR-FTIR spectra of Matrimid/HZIF-8 membranes.

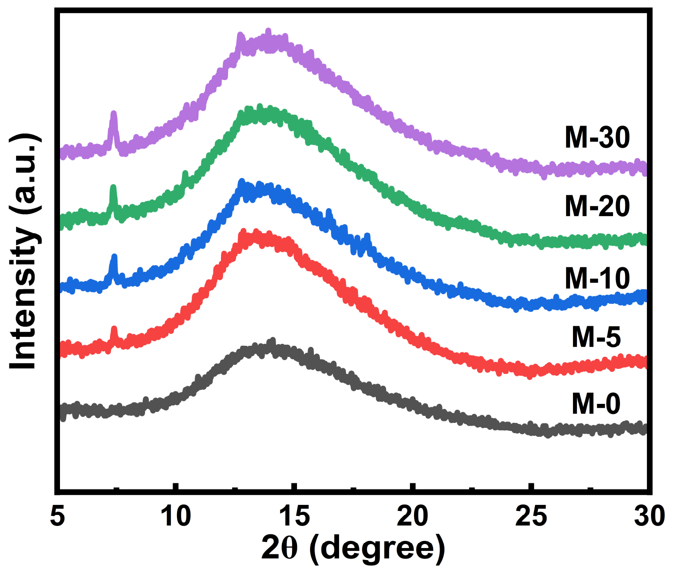

Supplementary Figure 27. XRD spectra of Matrimid/HZIF-8 membranes.

Supplementary Table 1. Specific surface area and pore volume and pore size of ZIF-8 and HZIF-8 nanoparticles.

Nanoparticle	BET surface area (m^2/g)	Total pore volume (ml/g)	H-K mode pore size (nm)
ZIF-8	1661.9	0.66	0.96
HZIF-8	265.9	0.23	0.74

Supplementary Table 2. The detailed chemical compositions from XPS analysis in ZIF-8 and HZIF-8.

Sample	Composition			
	Zn 2p	O 1s	N 1s	C 1s
ZIF-8	3.67	5.49	15.45	75.39
HZIF-8	1.68	21.71	3.62	72.99

Supplementary Table 3. PALS data of PIM-1, Z-5-1, and H-5-1 membranes.

Membrane	τ_3(ns)	I_3(%)	r_3(Å)	τ_4(ns)	I_4(%)	r_4(Å)
PIM-1	1.99±0.03	7.79±0.13	2.84±0.03	4.55±0.07	9.87±0.15	4.52±0.05
Z-5-1	2.18±0.02	9.84±0.18	3.01±0.02	4.72±0.08	10.17±0.21	4.61±0.07
H-5-1	1.97±0.03	7.94±0.10	2.82±0.03	4.75±0.07	9.91±0.19	4.62±0.07

Supplementary Table 4. Pure gas permeability and ideal selectivity of PIM-1 MMMs at 3.5 bar and 35 °C.

Loading of	Permeability (Barrer)			Ideal selectivity	
MOFs	N_2	CH_4	CO_2	CO_2/N_2	CO_2/CH_4
ZIF-8					
0(PIM-1)	309±15	416±20	6065±202	19.6±1.0	14.6±0.7
5-0.5	382±12	512±19	6917±223	18.1±1.0	13.5±0.6
5-1	822±21	1141±28	13564±347	16.5±0.8	11.9±0.6
5-3	2012±38	2687±43	22046±831	11.0±0.4	8.2±0.4
HZIF-8					
5-0.5	201±11	276±25	5442±149	27.1±0.9	19.7±0.6
5-1	309±13	441±19	8268±231	25.1±1.5	18.7±0.7
5-3	381±19	635±28	9024±237	23.7±1.2	16.7±1.0
5-5	568±14	1059±30	11057±305	19.5±1.1	13.4±0.6

Supplementary Table 5. Mixed-gas separation performance of PIM-1 and 0.1-ZIF-8/PIM-1 at 2 bar and 35 °C.

Membrane	Permeability (Barrer)				Selectivity	
	N_2	CH_4	CO_2[a]	CO_2[b]	CO_2/N_2	CO_2/CH_4
PIM-1	284±13	385±17	4786±164	4635±173	16.1±1.3	12.3±0.9
H-5-1	292±15	398±18	6495±192	6352±198	21.9±1.5	16.2±1.1

[a] obtained from the CO_2/N_2 mixture

[b] obtained from the CO_2/CH_4 mixture

Supplementary Table 6. Activation energy of permeation (E_p) for PIM-1 and H-5-1 membranes.

Gas	E_p (kJ/mol)	
	PIM-1	H-5-1
CO_2	4.00	-4.45
N_2	10.87	3.92
CH_4	8.27	2.56

Supplementary Table 7. Comparison of the CO_2 permeability and CO_2/gases selectivity of H-5-1 in this work with other reported MOF/PIM-1 MMMs.

MOF	Test conditions	CO_2 enhancement (%)	CO_2/N_2 selectivity enhancement (%)	CO_2/CH_4 selectivity enhancement (%)	Reference
ZIF-8	20 °C, 1 bar	10	-20	-	[1]
ZIF-8 (in situ)	35 °C, 3.5 bar	64	8.0	6.8	[2]
ZIF-67	30 °C, 2 bar	15	20	34	[3]
MIL-101A	35 °C, 3.5 bar	68	1	20	[4]
UiO-66	25 °C, 1 bar	79	0	-	[5]
UiO-66-NH_2	35 °C, 1 bar	32	7.1	1.2	[6]
UiO-66-NH_2/IL	20 °C, 1 bar	18	5.0	51	[7]
UiO-66-NH_2-M	25 °C, 4 bar	-6.0	71	95	[8]
HZIF-8	35 °C, 3.5 bar	36	28	29	This work

Supplementary Table 8. Comparison of the CO_2 permeability and CO_2/gases selectivity of MMMs in this work with other reported MMMs

Polymer	MOF	Test condition	CO_2 permeability (Barrer)	CO_2/N_2 selectivity	CO_2/CH_4 selectivity	Ref.
PIM-1	ZIF-8	20 °C, 1 bar	6820	17.9	13.4	[1]
PIM-1	ZIF-8 (in situ)	35 °C, 3.5 bar	6338	24.4	18.8	[2]
PIM-1	ZIF-67	30 °C, 2 bar	5206	24.2	16.8	[3]
PIM-1	HZIF-67	30 °C, 2 bar	7128	23.0	16.4	[9]
PIM-1	ZIF-S	30 °C, 2 bar	2805	24	21.1	[10]
PIM-1	UiO-66-NH_2-M	25 °C, 4 bar	2869	27.5	28.3	[8]
PIM-1	UiO-66	25 °C, 1 bar	13000	14.1	-	[5]
PIM-1	UiO-66-NH_2	35 °C, 1 bar	9420	15.6	12.3	[6]
PIM-1	UiO-66-NH_2/IL	20 °C, 1 bar	8283	22.5	12.3	[7]
PIM-1	ZIF-8	35 °C, 3.5 bar	13564	16.5	11.9	This work
PIM-1	HZIF-8	35 °C, 3.5 bar	8268	25.1	18.7	This work

[a] Estimated from gas flux through composite membranes

[b] obtained from the mixed gas test

Supplementary Table 9. Pure gas permeability and ideal selectivity of Matrimid-based MMMs at 3.5 bar and 35 °C.

Membrane	Permeability (Barrer)			Selectivity	
	CO_2	N_2	CH_4	CO_2/N_2	CO_2/CH_4
M-0	8.1±0.4	0.28±0.01	0.22±0.01	29.2±1.2	37.1±1.6
M-5	11.5±0.6	0.38±0.01	0.28±0.02	30.2±1.5	40.9±1.8
M-10	15.3±0.7	0.48±0.03	0.36±0.02	31.6±1.5	42.1±2.1
M-20	18.2±1.0	0.59±0.02	0.45±0.03	30.7±1.6	40.6±1.8
M-30	24.9±0.9	0.87±0.04	0.71±0.04	28.6±1.3	35.3±1.7

Supplementary Table 10. Comparison of separation performance of Matrimid/HZIF-8 membranes with other reported Matrimid/ZIF-8 membranes.

ZIF-8 content (wt%)	Test conditions	CO_2 enhancement (%)	CO_2/N_2 selectivity enhancement (%)	CO_2/CH_4 selectivity enhancement (%)	Ref.
20	35 °C, 3.5 bar	158	-3.4	0	[11]
30	35 °C, 3.5 bar	315	-37.9	-25.7	[11]
40	35 °C, 3.5 bar	1023	-65.5	-65.7	[11]
10	35 °C, 3.5 bar	89	8.2	13.5	This work
20	35 °C, 3.5 bar	125	5.1	9.4	This work
30	35 °C, 3.5 bar	207	1.4	3.2	This work

Supplementary Table 11. DSC data of Pebax/ZIF-8 and Pebax/HZIF-8 membranes

**Sample**	**T_g(°C)**	**PEO**		**PA6**	
		**T_{m1}(°C)**	**X_C(%)**	**T_m(°C)**	**X_C(%)**
Pebax	-50.9	17.9	30.8	204.0	27.3
ZIF-3%	-50.5	18.3	30.4	203.7	25.7
HZIF-3%	-49.7	19.0	27.7	203.8	24.7
ZIF-10%	-49.9	20.3	26.1	203.9	24.0
HZIF-10%	-47.6	19.3	25.7	203.8	21.5

Supplementary Table 12. Gas separation data of Pebax/ZIF-8 and Pebax/HZIF-8 membranes

Membrane	Permeability (Barrer)			Selectivity	
	CO_2	N_2	CH_4	CO_2/N_2	CO_2/CH_4
Pebax	115.2±3.1	2.4±0.1	7.5±0.4	48.6±1.5	15.4±1.0
ZIF-1%	127.7±4.1	2.6±0.1	7.7±0.5	49.3±2.0	16.6±1.2
ZIF-3%	153.0±5.7	3.2±0.2	9.4±0.7	48.4±1.9	16.3±1.3
ZIF-5%	169.5±5.5	3.6±0.1	10.5±0.7	47.0±1.5	16.1±1.1
ZIF-10%	177.2±6.0	4.5±0.3	12.2±0.8	39.2±1.3	14.6±0.9
HZIF-1%	147.5±4.5	2.7±0.2	7.3±0.5	54.2±2.5	20.1±1.6
HZIF-3%	197.4±6.1	3.0±0.3	7.4±0.3	64.9±2.3	26.7±1.8
HZIF-5%	181.6±5.9	2.8±0.1	6.7±0.4	65.3±2.5	27.3±1.3
HZIF-10%	120.5±3.2	1.9±0.1	4.4±0.2	64.7±2.0	27.3±1.1

Supplementary References

1. Bushell A.F.*, et al.* Gas permeation parameters of mixed matrix membranes based on the polymer of intrinsic microporosity PIM-1 and the zeolitic imidazolate framework ZIF-8. *J Membr Sci* **427**, 48-62 (2013).

2. He S.*, et al.* Symbiosis-inspired de novo synthesis of ultrahigh MOF growth mixed matrix membranes for sustainable carbon capture. *Proc Natl Acad Sci U S A* **119**, e2114964119 (2022).

3. Wu X.*, et al.* Nanoporous ZIF-67 embedded polymers of intrinsic microporosity membranes with enhanced gas separation performance. *J Membr Sci* **548**, 309-318 (2018).

4. Khdhayyer M.*, et al.* Mixed matrix membranes based on MIL-101 metal–organic frameworks in polymer of intrinsic microporosity PIM-1. *Sep Purif Technol* **212**, 545-554 (2019).

5. Prasetya N. & Ladewig B.P. An insight into the effect of azobenzene functionalities studied in UiO-66 frameworks for low energy CO_2 capture and CO_2/N_2 membrane separation. *J Mater Chem A* **7**, 15164-15172 (2019).

6. Wang Z.*, et al.* Polymers of intrinsic microporosity/metal–organic framework hybrid membranes with improved interfacial interaction for high-performance CO_2 separation. *J Mater Chem A* **5**, 10968-10977 (2017).

7. Lu J.*, et al.* Preparation of Amino-Functional UiO-66/PIMs Mixed Matrix Membranes with [bmim][Tf_2N] as Regulator for Enhanced Gas Separation. *Membranes (Basel)* **11**, 35 (2021).

8. Ghalei B.*, et al.* Enhanced selectivity in mixed matrix membranes for CO_2 capture through efficient dispersion of amine-functionalized MOF nanoparticles. *Nature Energy* **2**, 17086 (2017).

9. Wu X.*, et al.* Accelerating CO_2 capture of highly permeable polymer through incorporating highly selective hollow zeolite imidazolate framework. *AIChE J* **66**, e16800 (2019).

10. Ye C.*, et al.* Incorporating nano-sized ZIF-67 to enhance selectivity of polymers of intrinsic microporosity membranes for biogas upgrading. *Chem Eng Sci* **216**, 115497 (2020).

11. Jiang X.*, et al.* Aqueous One-Step Modulation for Synthesizing Monodispersed ZIF-8 Nanocrystals for Mixed-Matrix Membrane. *ACS Appl Mater Interfaces* **13**, 11296-11305 (2021).
